# Supplementary material for: Enterococcus faecium secreted antigen A generates muropeptides to enhance host immunity and limit bacterial pathogenesis
Source: eLife. 2019 Apr 10;8:e45343. doi: 10.7554/eLife.45343 (PMC6483599; doi:10.7554/eLife.45343)
Supplement: Supplementary file 1. — (a) Peak numbers refer to Figure 2-b. (b) GM, disaccharide (GlcNAc-MurNAc); 2 GM, disaccharide-disaccharide (GlcNAc-MurNAc-GlcNAc-MurNAc); 3 GM, disaccharide-disaccharide-disaccharide (GlcNAc-MurNAc-GlcNAc-MurNAc- GlcNAc-MurNAc); GM-Tri, disaccharide tripeptide (L-Ala-D-iGln-L-Lys); GM-Tetra, disaccharide tetrapeptide (L-Ala-D-iGln-L-Lys-D-Ala); GM-Penta, disaccharide pentapeptide (L-Ala-D-iGln-L-Lys-D-Ala -D-Ala). [file elife-45343-supp1.docx]

**Supplementary Table 1. Molecular mass and composition of muropeptides from *E. faecium.***

| Peak^a^ | RT (min) | calculated  [M+H]^+^ | observed [M+H]^+^ | Proposed structure^b^ |
| --- | --- | --- | --- | --- |
| 1 | 16.2 | 826.40 | 826.40 | GM-tri |
| 2 | 21.6 | 897.44 | 897.44 | GM-tetra |
| 3 | 22.7 | 940.45 | 940.45 | GM-tri (Asn)^d^ |
| 4 | 24.5 | 941.43 | 941.43 | GM-tri (Asp) ^d^ |
| 5 | 26.7 | 1011.48 | 1011.48 | GM-tetra (Asn) |
| 6 | 27.6 | 1082.52 | 1082.52 | GM-penta (Asx) |
| 7 | 30.6 |  | 1453.72 | ND^c^ |
| 8 | 32.7 | 1819.88 | 1819.88 | 2GM-tri (Asx) – tetra |
| 9 | 35.2 | 1933.92 | 1933.92 | 2GM-tri (Asn) - tetra (Asn)^d^ |
| 10 | 36.3 | 1934.90 | 1934.90 | 2GM-tri (Asx) - tetra (Asx)^d^ |
| 11 | 37.9 | 2004.96 | 2004.96 | 2GM-tetra (Asn) - tetra (Asn)^d^ |
| 12 | 38.9 | 2006.93 | 2006.94 | 2GM-tetra (Asp) - tetra (Asp)^d^ |
| 13 | 41.3 | 2928.38 | 2928.39 | 3GM-tetra (Asx) - tetra (Asx) – tri (Asx)^d^ |
| 14 | 41.9 | 2929.36 | 2929.38 | 3GM-tetra (Asx) - tetra (Asx) – tri (Asx)^d^ |
| 15 | 43.1 | 3000.44 | 3000.44 | 3GM-tetra (Asx) - tetra (Asx) – tetra (Asx) |

a. Peak numbers refer to Figure 2-b.

b. GM, disaccharide (GlcNAc-MurNAc); 2GM, disaccharide-disaccharide (GlcNAc-MurNAc-

GlcNAc-MurNAc); 3GM, disaccharide-disaccharide-disaccharide (GlcNAc-MurNAc-

GlcNAc-MurNAc- GlcNAc-MurNAc); GM-Tri, disaccharide tripeptide (L-Ala-D-iGln-L-Lys);

GM-Tetra, disaccharide tetrapeptide (L-Ala-D-iGln-L-Lys-D-Ala); GM-Penta, disaccharide

pentapeptide (L-Ala-D-iGln-L-Lys-D-Ala -D-Ala).
