# Supplementary material for: Enterococcus faecium secreted antigen A generates muropeptides to enhance host immunity and limit bacterial pathogenesis
Source: eLife. 2019 Apr 10;8:e45343. doi: 10.7554/eLife.45343 (PMC6483599; doi:10.7554/eLife.45343)
Supplement: Supplementary file 2. — (a) Peak numbers refer to Figure 2-b. (b) GM, disaccharide (GlcNAc-MurNAc); 2 GM, disaccharide-disaccharide (GlcNAc-MurNAc-GlcNAc-MurNAc); 3 GM, disaccharide-disaccharide-disaccharide (GlcNAc-MurNAc-GlcNAc-MurNAc- GlcNAc-MurNAc); GM-Tri, disaccharide tripeptide (L-Ala-D-iGln-L-Lys); GM-Tetra, disaccharide tetrapeptide (L-Ala-D-iGln-L-Lys-D-Ala); GM-Penta, disaccharide pentapeptide (L-Ala-D-iGln-L-Lys-D-Ala -D-Ala). (c) ND: Precise structure unknown. d. The assignment of the amide and the hydroxyl functions to either peptide stem is arbitrary. [file elife-45343-supp2.docx]

**Supplementary Table 2. Molecular mass and composition of muropeptides from *E. faecalis* and *E. faecalis-sagA*.**

| Peak^a^ | RT (min) | calculated  [M+H]^+^ | observed [M+H]^+^ | Proposed structure^b^ |
| --- | --- | --- | --- | --- |
| 1 | 25.3 | 897.44 | 897.44 | GM-tetra |
| 2 | 27.9 | 968.48 | 968.48 | GM-tri (AA) |
| 3 | 29.8 | 969.46 | 969.46 | GM-tri (AA), Gln/Glu^d^ |
| 4 | 31.9 | 1110.55 | 1110.55 | GM-penta (AA) |
| 5 | 33.8 | 1111.53 | 1111.53 | GM-penta (AA), Gln/Glu^d^ |
| 6 | 41.5 |  | 2070.95 | ND^c^ |
| 7 | 43.1 | 1989.98 | 1989.98 | 2GM-tri (AA) - tetra (AA) |
| 8 | 43.8 |  | 2190.12 | ND^c^ |
| 9 | 44.3 | 1991.95 | 1991.97 | 2GM-tri (AA) - tetra (AA), Gln/Glu x2^d^ |
| 10 | 45.6 | 2132.06 | 2133.06 | 2GM-penta (AA) - tetra (AA) |
| 11 | 47.0 | 2134.03 | 2134.05 | 2GM-penta (AA) - tetra (AA), Gln/Glu x2^d^ |
| 12 | 47.6 | 2134.03 | 2134.05 | 2GM-penta (AA) - tetra (AA), Gln/Glu x2^d^ |
| 13 | 51.0 | 3012.47 | 3012.49 | 3GM-tri (AA) - tetra (AA) - tetra (AA), Gln/Glu |
| 14 | 52.8 | 3154.55 | 3154.57 | 3GM-penta (AA) - tetra (AA) - tetra (AA), Gln/Glu^d^ |
| 15 | 53.8 | 3155.53 | 3155.55 | 3GM-penta (AA) - tetra (AA) - tetra (AA), Gln/Glu x2^d^ |
| 16 | 54.2 | 3155.53 | 3155.55 | 3GM-penta (AA) - tetra (AA) - tetra (AA), Gln/Glu x2^d^ |
| 17 | 55.9 | 4033.98 | 4033.99 | 4GM-tri (AA) - tetra (AA) - tetra (AA) - tetra (AA), Gln/Glu^d^ |
| 18 | 57.3 | 4176.05 | 4176.07 | 4GM-penta (AA) - tetra (AA) - tetra (AA) - tetra (AA), Gln/Glu^d^ |
| 19 | 58.4 | 4177.03 | 4177.05 | 4GM-penta (AA) - tetra (AA) - tetra (AA) - tetra (AA), Gln/Glu x2^d^ |
| 20 |  | 4178.02 | 4178.04 | 4GM-penta (AA) - tetra (AA) - tetra (AA) - tetra (AA), Gln/Glu x3^d^ |

a. Peak numbers refer to Figure 2-b.

b. GM, disaccharide (GlcNAc-MurNAc); 2GM, disaccharide-disaccharide (GlcNAc-MurNAc-

GlcNAc-MurNAc); 3GM, disaccharide-disaccharide-disaccharide (GlcNAc-MurNAc-

GlcNAc-MurNAc- GlcNAc-MurNAc); GM-Tri, disaccharide tripeptide (L-Ala-D-iGln-L-Lys);

GM-Tetra, disaccharide tetrapeptide (L-Ala-D-iGln-L-Lys-D-Ala); GM-Penta, disaccharide

pentapeptide (L-Ala-D-iGln-L-Lys-D-Ala -D-Ala).

c. ND: Precise structure unknown.

d. The assignment of the amide and the hydroxyl functions to either peptide stem is arbitrary.
