# Supplementary material for: Enterococcus faecium secreted antigen A generates muropeptides to enhance host immunity and limit bacterial pathogenesis
Source: eLife. 2019 Apr 10;8:e45343. doi: 10.7554/eLife.45343 (PMC6483599; doi:10.7554/eLife.45343)
Supplement: Supplementary file 3. — All sequencing data are available from GenBank under accession number CP025022, CP025020, and CP025021 for Enterococcus faecium Com15, Enterococcus faecalis OG1RF, and Enterococcus faecalis OG1RF-sagA. [file elife-45343-supp3.docx]

**Supplementary Table 3. List of genes that have synonymous and non-synonymous mutations in *E. faecalis*-*sagA* compared with *E. faecalis*.**

| CDS | non-synonymous | synonymous | frequency range |
| --- | --- | --- | --- |
| yqeH CDS | 3 | 3 | 25.90-42.30% |
| uppP CDS | 3 | 3 | 29.20-41.70% |
| ubiA2 CDS | 0 | 1 | 27.60% |
| transglycosylase-associated protein CDS | 1 | 0 | 34.80% |
| tkt CDS | 0 | 1 | 97.10% |
| srlE CDS | 1 | 0 | 26.60% |
| SNARE associated protein CDS | 3 | 0 | 25.80-30.0% |
| selD CDS | 0 | 1 | 40.00% |
| RsmD family RNA methyltransferase CDS | 0 | 1 | 95.20% |
| rplD CDS | 0 | 1 | 28.30% |
| radA CDS | 3 | 1 | 26.80-33.30% |
| pyrDB CDS | 1 | 0 | 28.30% |
| purL CDS | 0 | 1 | 26.70% |
| protein of hypothetical function UPF0052 and CofD CDS | 1 | 1 | 29.60-36.40% |
| ppx3 CDS | 0 | 1 | 100.00% |
| potassium uptake protein CDS | 0 | 1 | 87.50% |
| permease protein CDS | 8 | 0 | 25.90-50.00% |
| penicillin-binding protein 4 CDS | 0 | 1 | 96.30% |
| oxidoreductase CDS | 0 | 1 | 76.70% |
| NADPH-dependent FMN reductase domain protein CDS | 0 | 1 | 97.20% |
| mtlD CDS | 1 | 0 | 100.00% |
| mtaD2 CDS | 0 | 1 | 100.00% |
| mraY CDS | 1 | 0 | 33.30% |
| menF CDS | 0 | 1 | 86.70% |
| Lysyl-tRNA synthetase (class II) CDS | 8 | 0 | 26.30-36.10% |
| lipase CDS | 0 | 1 | 30.60% |
| iolG2 CDS | 1 | 0 | 93.80% |
| gor CDS | 4 | 0 | 25.50-31.50% |
| glucuronyl hydrolase CDS | 1 | 1 | 31.30-37.50% |
| gltX CDS | 1 | 0 | 26.80% |
| fhs CDS | 1 | 0 | 28.60% |
| fabG2 CDS | 0 | 1 | 92.30% |
| dnaG CDS | 0 | 1 | 100.00% |
| dinB CDS | 0 | 1 | 100.00% |
| DAACS family dicarboxylate/amino acid:cation symporter CDS | 3 | 2 | 26.50-43.20% |
| atpI CDS | 1 | 0 | 31.80% |
| atoB CDS | 2 | 2 | 31.80-57.10% |
| aspC2 CDS | 1 | 0 | 31.30% |
| asd CDS | 0 | 1 | 95.50% |
| arcC2 CDS | 1 | 2 | 28.30-93.80% |
| acyltransferase CDS | 1 | 0 | 100.00% |
| accA CDS | 3 | 0 | 32.0-38.50% |

All sequencing data are available from GenBank under accession number CP025022, CP025020, and CP025021 for *Enterococcus faecium* Com15, *Enterococcus faecalis* OG1RF, and *Enterococcus faecalis* OG1RF-*sagA*.
