# Supplementary material for: Enterococcus faecium secreted antigen A generates muropeptides to enhance host immunity and limit bacterial pathogenesis
Source: eLife. 2019 Apr 10;8:e45343. doi: 10.7554/eLife.45343 (PMC6483599; doi:10.7554/eLife.45343)
Supplement: Supplementary file 4. — (a) Peak numbers refer to Supplementary file 5e. (b) GM, disaccharide (GlcNAc-MurNAc); GM-di, disaccharide dipeptide (L-Ala-D-iGln); GM-Tri, disaccharide tripeptide (L-Ala-D-iGln-L-Lys); GM-Tetra, disaccharide tetrapeptide (L-Ala-D-iGln-L-Lys-D-Ala). [file elife-45343-supp4.docx]

**Supplementary Table 4. Molecular mass and composition of enzymatic products from incubation of *E. faecalis* PG with purified SagA-NlpC/p60 domain.**

| Peak^a^ | RT (min) | calculated [M+H]^+^ | observed [M+H]^+^ | Proposed structure^b^ |
| --- | --- | --- | --- | --- |
| a | 12.0 | 431.26 | 431.26 | Tri (AA) |
| b | 12.1 | 698.31 | 698.31 | GM-di (AA) |
| c | 34.4 | 1310.69 | 1310.69 | GM-tri-di (AA x2) |
| d | 37.4 | 1452.77 | 1452.77 | GM-tetra-tri (AA x2) |
| e | 47.3 | 2474.27 | 2474.27 | GM-tetra-GM-tetra-tri (AA x3) |

a. Peak numbers refer to Supplementary Figure 5e.

b. GM, disaccharide (GlcNAc-MurNAc); GM-di, disaccharide dipeptide (L-Ala-D-iGln); GM-

Tri, disaccharide tripeptide (L-Ala-D-iGln-L-Lys); GM-Tetra, disaccharide tetrapeptide (L-Ala-

D-iGln-L-Lys-D-Ala).
