# Supplementary material for: Enterococcus faecium secreted antigen A generates muropeptides to enhance host immunity and limit bacterial pathogenesis
Source: eLife. 2019 Apr 10;8:e45343. doi: 10.7554/eLife.45343 (PMC6483599; doi:10.7554/eLife.45343)
Supplement: Supplementary file 5. — (a) One crystal was used to determine structure. (b) Values in parentheses are for highest resolution shell. [file elife-45343-supp5.docx]

**Supplementary Table 5. Crystallographic statistics.**

|  | SagA-NlpC/p60^a^ |
| --- | --- |
| PDB entry | 6B8C |
| Data collection |  |
| Beamline | NSLS-II AMX |
| Space group | P4 3 2 |
| Cell dimensions |  |
| a, b, c (Å) | 100.38, 100.38, 100.38 |
| α, β, γ (°) | 90, 90, 90 |
| Wavelength (Å) | 0.9793 |
| Resolution range (Å) | 50.00 – 2.40 (2.49 - 2.40)^b^ |
| No. of total reflections | 92864 (3032) |
| No. of unique reflections | 7166 (645) |
| Completeness (%) | 99.3 (93.2) |
| I / σ (I) | 32.4 (0.52) |
| CC_1/2_ | 0.984 (0.334) |
| R_merge_ | 0.095 (1.579) |
| R_meas_ | 0.099 (1.753) |
| R_pim_ | 0.026 (0.734) |
| Multiplicity | 13.0 (4.7) |
|  |  |
| Refinement statistics |  |
| Resolution range (Å) | 50.00 – 2.40 |
| No. of reflections | 7155 (634) |
| reflections used for Rfree | 717 (64) |
| R_work_ / R_free_ | 0.24 / 0.27 |
| No. atoms | 886 |
| macromolecules | 878 |
| Ligand/ion | 0 |
| Water | 8 |
| R.m.s deviations |  |
| Bond lengths (Å) | 0.003 |
| Bond angles (°) | 0.61 |
| Average B-factors |  |
| macromolecules | 75.75 |
| Ramachandran favored (%) | 97.41 |
| Ramachandran outliers (%) | 0.00 |
| Rotamer outliers (%) | 3.45 |
| Clashscore | 4.66 |

^a^One crystal was used to determine structure

^b^Values in parentheses are for highest resolution shell
