# Supplementary material for: Enterococcus faecium secreted antigen A generates muropeptides to enhance host immunity and limit bacterial pathogenesis
Source: eLife. 2019 Apr 10;8:e45343. doi: 10.7554/eLife.45343 (PMC6483599; doi:10.7554/eLife.45343)
Supplement: Supplementary file 6. — The structural alignment was performed by the DALI server (Holm and Sander, 1995). For structures with multiple chains/models, only results for the first structure with the highest Z-score are shown. No. of residues: the number of residues present in the model used for comparison; Seq id: percentage sequence identity of the pairwise structural alignment. [file elife-45343-supp6.docx]

**Supplementary Table 6. Structural comparisons of SagA-NlpC/p60 domain with structurally similar homologs determined by DALI server^a^**

| Name | Description | Organism | PDB (chain) | RMSD (Å) | No. of residues | Z score | Seq ID (%) |
| --- | --- | --- | --- | --- | --- | --- | --- |
| YkfC | NlpC/p60 | *Bacillus*  *cereus* | 3H41 (A) | 1.6 | 307 | 19.3 | 30 |
| NpPCP | Endopeptidase | *Nostoc punctiforme* | 2EVR (A) | 2.0 | 222 | 17.0 | 26 |
| AvPCP | NlpC/p60 | *Anabaena variabilis* | 2HBW (A) | 2.0 | 220 | 17.0 | 26 |
| CwlT | NlpC/p60 | *Staphylococcus aureus* | 4FDY (A) | 2.0 | 295 | 16.8 | 35 |
| Spr | Lipopeptide | *Escherichia*  *coli* | 2K1G (A) | 2.0 | 129 | 16.1 | 28 |
| RipA | Endopeptidase | *Mycobacterium tuberculosis* | 3NE0 (A) | 1.9 | 208 | 14.2 | 34 |
| LysM | NlpC/p60 | *Thermus thermophilus* | 4XCM (B) | 1.9 | 218 | 14.0 | 30 |

^a^The structural alignment was performed by the DALI server (Holm & Sander, 1995). For structures with multiple chains/models, only results for the first structure with the highest Z-score are shown. No. of residues: the number of residues present in the model used for comparison; Seq id: percentage sequence identity of the pairwise structural alignment.
