# Supplementary material for: Enterococcus faecium secreted antigen A generates muropeptides to enhance host immunity and limit bacterial pathogenesis
Source: eLife. 2019 Apr 10;8:e45343. doi: 10.7554/eLife.45343 (PMC6483599; doi:10.7554/eLife.45343)
Supplement: Supplementary file 7. — MM-GBSA calculations were carried out using the Prime_MM-GBSA utility. [file elife-45343-supp7.docx]

**Supplementary Table 7. Predicted binding free energies of highest-scoring poses of docked GlcNAc-MurNAc-L-Ala-D-isoGln-L-Lys-D-Ala as generated with MM-GBSA^a^.**

| Pose | predicted ΔG (kcal/mol) | Pose | predicted ΔG (kcal/mol) | Pose | predicted ΔG (kcal/mol) |
| --- | --- | --- | --- | --- | --- |
| 1 | *-54.2* | 21 | -36.0 | 41 | -23.8 |
| 2 | -48.4 | 22 | -17.0 | 42 | -17.4 |
| 3 | -54.8 | 23 | -17.1 | 43 | -45.1 |
| 4 | -57.5 | 24 | -33.4 | 44 | -27.1 |
| 5 | -53.9 | 25 | -30.5 | 45 | -6.6 |
| 6 | -18.4 | 26 | -45.9 | 46 | -15.1 |
| 7 | -58.4 | 27 | -37.8 | 47 | -21.5 |
| 8 | -40.5 | 28 | -23.1 | 48 | -21.9 |
| 9 | -39.4 | 29 | -36.0 | 49 | -42.0 |
| 10 | -30.4 | 30 | -32.8 | 50 | 30.0 |
| 11 | -30.8 | 31 | -49.5 |  |  |
| 12 | -52.7 | 32 | -33.7 |  |  |
| 13 | -20.5 | 33 | -26.1 |  |  |
| 14 | -41.1 | 34 | -22.6 |  |  |
| 15 | -14.6 | 35 | -23.5 |  |  |
| 16 | -14.8 | 36 | -30.1 |  |  |
| 17 | -44.9 | 37 | -33.1 |  |  |
| 18 | -42.7 | 38 | -25.9 |  |  |
| 19 | -38.0 | 39 | -74.2 |  |  |
| 20 | -42.0 | 40 | -51.1 |  |  |

a. MM-GBSA calculations were carried out using the Prime_MM-GBSA utility.
